# Science- and risk-based strategy to qualify prefillable autoclavable syringes as primary packaging material

**DOI:** 10.1136/ejhpharm-2020-002333

**Published:** 2021-01-27

**Authors:** Karin Larmené-Beld, Rommert Wijnsma, Albert Kuiper, Stefan van Berkel, Henri Robben, Katja Taxis, Henderik Frijlink

**Affiliations:** 1 Clinical Pharmacy, Isala Hospitals, Zwolle, The Netherlands; 2 Unit of PharmacoTherapy, Epidemiology & PharmacoEconomics, Groningen Research Institute of Pharmacy, University of Groningen, Groningen, The Netherlands; 3 Department of Pharmaceutical Technology and Biopharmacy, University of Groningen, Groningen, The Netherlands

**Keywords:** drug manufacturing / preparation / compounding, Good Manufacturing Practice (GMP), sterile production, drug stability, validation preparation process

## Abstract

**Objectives:**

To develop a science and risk based strategy to qualify a prefillable autoclavable cyclic olefin polymer (COP) syringe as a container for multiple drug products in a hospital pharmacy setting

**Methods:**

Different extraction studies were performed with different solution characteristics: phosphate buffer batches (Na_2_HPO_4_ / NaH_2_PO_4_ in NaCl 0.9%) at different pH values, NaCl 0.9% batches, isopropyl alcohol (IPA) 5% in water and batches with Water For Injections (WFI) at different pH values. The filled syringes were terminally sterilised. The syringes were stored at room temperature (20°C±5°C).

Following different monographs of the European Pharmacopoeia several tests were performed on the different batches. Analyses were performed at t=0, 1, 2, 3, 4, 5, 6, 9, 12, 18, 24 and 36 months for the general tests. For the subvisible particles, sterility and closure integrity a bracketing scheme was applied during 36 months.

**Results:**

Low levels of extractables were measured for the different solutions. The test for subvisible particles, sterility and closure integrity all met predefined requirements. In the 5mL and 50mL syringes different concentrations of silicon were measured. Overall higher silicon concentrations were measured for the 50mL syringes.

**Conclusions:**

The chosen strategy for the qualification program provided an adequate understanding about the extractables that could leak from the syringes. The cyclic olefin polymer syringes including stopper and tip cap were found to be suitable as primary packaging materials for the production of water based products.

## Introduction

Medication safety in hospitals can be improved by providing ready to use/ ready to administer (RTU/ RTA) parenteral medications produced by the hospital pharmacy or industry as recommended by The Joint Commission International Accreditation Standards for Hospitals.^1^ In the Netherlands many hospitals facilitate this by aseptic filling of polypropylene single use syringes. These syringes have a shelf life of 31 days in the refrigerator according to - hospital pharmacy GMP guidelines (Dutch).^2^ Major problems related to this practice are: (1) the aseptic process with a lack of sterilisation in the final container; (2) the limited shelf-life; (3) the refrigerator capacity that is needed for storage, both in the pharmacy and on the ward; (4) the need for a cold chain for the entire production and distribution process including the need for a validation of this process; (5) the lack of the qualifications of the syringes as containers and (6) the limited capabilities of upscaling the production. A terminally sterilised product is preferred because it provides a much higher level of quality assurance.^3^ To produce prefillable syringes suitable for final sterilisation, the BD Sterifill Advance syringe can be used. This is a cyclic olefin polymer (COP) syringe which can be terminally sterilised. Although all individual components comply with the regulatory demands; e.g. for plastics; USP 661, JP 61, for rubber: USP381, Ph. Eur 3.2.9, JP 59, ISO 8871–1 and for biocompatibility/ toxicological USP 87, EMA, 88, ISP 10993, TSE Ph. Eur. 5.2.8, it is the responsibility of the drug product manufacturer to ensure that the new packaging material (being a combination of compliant materials) does not adversely affect patient safety and does meet predefined product quality requirements.^4^ However, so far no general monographs are available to test the compatibility of a new product in COP syringes. Ph. Eur. Monograph 3.2.8 “sterile single use plastic syringes” is only applicable for syringes for “immediate use”. The European Medicine Agency (EMA) has published a guideline on plastic immediate packaging materials in) which a decision tree is given for a practical approach when the plastic is not described in a general monograph of the Ph. Eur.^4^ The monographs Ph. Eur. 3.2.2 Plastic containers and closures for pharmaceutical use/ 3.2.9. rubber closure for containers for aqueous parenteral preparations, for powders and for freeze dried powders state that the plastic container and closure chosen for any particular product should be such that:

- The ingredients of the product in contact with the plastic material and closure are not significantly adsorbed on its surface and do not significantly migrate into or through the plastic/ closure to an extent sufficient to affect the product adversely,
- The plastic material or closure does not release substances in quantities sufficient to affect the stability of the product or to present a risk of toxicity.

In this paper we will give an overview of our strategy and results to qualify a COP terminally sterilised syringe as a container for multiple drug products in a hospital pharmacy setting.

## Materials and methods

### Selection of candidates to be produced as prefilled syringes

An analysis of the parenteral products which are regularly used in hospitals, revealed a range of products which would be interesting for larger scale production in prefilled syringes. The analysis started by identifying injections that were frequently administered (high volume) and that were always given in standardised dosages. The outcome of the analysis resulted in a set of products of water soluble drugs with a pH range from 3 to 9.3 in volumes of 2, 5, 10 or 50 mL, filled in either 5 (both 2 and 5 mL filling volume), 10 or 50 mL syringes. Eligible products included for example metoclopramide HCl, morphine HCl, potassium chloride and midazolam. From literature it is known that, in particular, the pH and the polarity of the solvent are essential variables that determine the egress of possible extractables and leachables from the syringe.^5 6^ For this reason, it was decided to limit the first qualification to aqueous solvents covering a pH range from 2 to 11 and to investigate the effect of the addition of an apolar solvent to the solution.

### The syringes

The sterilised COP syringe system was a BD Medical, Pharmaceutical System (BD) Sterifill Advance 50 mL syringe with a polypropylene/ butyl rubber tip cap and a butyl rubber stopper and a Sterifill 5 mL syringe with an elastomer tip cap and a butyl rubber stopper. See table 1 for specific components and details. Batch to batch quality of the individual components was guaranteed by the technical dossier, qualifications specification and the certificate of analysis of batch numbers of the manufacturer and meet all necessary requirements of primary packaging material for medicinal products.^7 8^ The 5 mL syringe filled with 5 mL solvent was the worst case scenario because of the highest contact surface to product volume ratio. But both syringes were tested in the study because of the transitional situation of the Sterifill to the Sterifill Advance system.

**Table 1:** Specification primary packaging material

Component	5 mL syringe	50 mL syringe
Syringe barrel	BD Crystal Clear Polymer; (polycycloolefine) Lubrication: silicone coating	BD Crystal Clear Polymer (polycycloolefine) Lubrication: silicone coating
Plunger stopper – Silicone	SBR rubber – DC 360	FM457 butyl rubber – Rhodia 70 047
Tip cap	Luer lok: Thermoplastic elastomer blend	S-Lok; plastic part: Polypropylene Rubber part: Butyl rubber.

An extractables study report from the manufacturer was available with a list of potential extractables. Mostly acids in low concentrations occur as extracts from the barrel, stopper and tip cap and also a couple of unknown organic compounds, hexane and toluene from the stopper. All measured components were within the acceptance limits of regulatory guidelines.^7 8^ Based on this information and the intended drug products we decided to qualify the container as primary packaging material according to the extraction study described below.

### Extraction studies

Extraction studies were conducted with isotonic buffer solutions covering the desired range of pH values. In order to determine the influence of the pH on extractables, phosphate buffer batches (Na_2_HPO_4_ / NaH_2_PO_4_ in NaCl 0.9%) were produced with a pH of 2.0, 5.8, 8.0 and 11.4. In addition, NaCl 0.9% was used to investigate whether possible extractables and leachables lead to a pH change in the solvent. Furthermore, a batch of isopropyl alcohol (IPA) 5% in water was made to investigate which extractables and leachables migrated when an organic solvent was used. IPA was also used as a model substance for apolar drugs that may cause (additional) migration of apolar extractables and leachables. Finally, separate batches with Water For Injection (WFI) at different pH values (2–5 and 8–11) were produced for measuring silicon levels in the syringes.

The process conditions for all of the batches were the same as during regular production. The filled syringes were all terminally sterilised, with the final sterilisation step carried out at 121°C for 15 min. The storage conditions were the same as the final product storage conditions including storage temperature (20°C±5°C), product contact surface, and extraction volume.

Following different monographs of the European Pharmacopoeia 11 tests were performed on the different batches (table 2). Analyses were performed at t=0, 1, 2, 3, 4, 5, 6, 9, 12, 18, 24 and 36 months for the general chemistry tests (test 1–7). For the subvisible particles, sterility and closure integrity a bracketing scheme was applied during 36 months.

**Table 2:** Tests performed in the extraction study

Test	Monograph	Description/ acceptance limit
1. Clarity and degree of opalescence of the solution	Ph. Eur. 2.2.1.^14^	The clarity of the solution is the same as that of *water R*. The absence of any particles or inhomogeneties in a solution results in a clear solution
2. Degree of colouration of the solution	Ph. Eur. 2.2.2^15^	Examination of the degree of colouration of the solution in the range brown-yellow-red
3. pH of the solution	Ph. Eur. 2.2.3	Measurement of the pH
4. Absorbance (extractables and leachables)	Ph. Eur 3.2.2.1^16^	The absorbance of the solution was measured from 230nm to 360nm. At these wavelengths the absorbance should not be not greater than 0.20
5. Reducing substances	Ph. Eur. 3.2.2.1^16^	To 20.0 mL of solution S add 1 mL of dilute sulfuric acid R and 20.0 mL of 0.002M potassium permanganate. Boil for 3 min. Cool immediately. Add 1 g of potassium iodide R and titrate immediately with 0.01 M sodium thiosulfate, using 0.25 mL of starch solution R as indicator. Carry out a titration using 20.0 mL of the blank. The difference between the titration volumes is not greater than 1.5 mL.
6. Transparency	Ph. Eur 3.2.2.1^16^	Fill a container previously used for the preparation of solution S with a volume equal to the nominal capacity of the primary opalescent suspension (2.2.1) diluted 1 in 200 for a container made from polyethylene or propylene and 1 in 400 for other containers. The cloudiness of the suspension is perceptible when viewed through the container and compared with a similar container filled with water R.
7. Weight loss	Local hospital standard	≤2% wt loss compared with the initial weight.
8. Subvisible particles	Ph. Eur. 2.9.19^17^	According to method 1. Light obscuration particle count test. The solution complies with the test if the average number of particles present in the units tested does not exceed 6000 per container equal to or greater than 10 µm and does not exceed 600 per container equal to or greater than 25 µm.
9. Closure integrity test	Ph. Eur. 3.2.9./ manipulated^18^	A dye immersion test with 0.1% methylene blue. Immerse the syringes in a 1 g/L solution of methylene blue and reduce the external pressure by 27 kPa for 10 min. Restore atmospheric pressure and leave the vials immersed for 30 min. Rinse the outside of the syringes. None of the vials contains any trace of coloured solution
10. Sterility	Ph. Eur. 2.6.1^9^	Sterility test with the membrane filtration method, with fluid thioglycollate medium (FTM) and Tryptic Soy Broth (Soybean-Casein Digest Medium) medium (TSB). Incubation during 14 days
11. Silicon*	–	ICP- MS method (see method section for description)

*Silicon concentrations were only measured in the WFI batches.

### Closure integrity test

The Ph. Eur. Monograph 3.2.9 describes a self-sealing test. This test was developed to investigate the closure integrity of the seal of multidose containers. However, in this study the test was manipulated to test the closure integrity of de COP syringes.

#### Method

A total of 10 syringes were immersed into the 0.1% methylene blue solution in a well closed stainless-steel vessel. The atmospheric pressure in the vessel was reduced to 27 kPa for 10 min. Finally, the atmospheric pressure was restored. In case of an adequately closed syringe, the pressure inside the syringe is unchanged. In case a leakage occurred, the pressure in the headspace of the syringe was reduced, and after the atmospheric pressure was restored, the 0.1% methylene blue solution penetrated into the syringe within 30 min. Visual inspection of the contents of the syringes (comparing against water) was used to detect leakage. The test was passed when none of the syringes showed a trace of blue colour.

#### Method validation

Method validation has been performed using a positive and negative control. Due to the hardness of the COP material a capillary tube with a predefined diameter was pushed through the stopper of the syringe. In pre-validation tests of the closure integrity test, capillaries with different internal diameters were used to define the limit of discriminatory power of the test. During the validation test the detection of the leakage was performed by visual inspection of the contents of the syringes (comparing against water). The test was passed when none of the syringes showed a trace of blue colour. The capillair with 170 µm internal diameter was the smallest capillair with blue colorisation of the solution for the positive control. The positive control syringe contains a tube with an outer diameter of 500 µm an inner diameter of 170 µm. The negative control syringe contained a Remanium (CoCr alloy) wire with a similar outer diameter. The method was validated with 10 syringes of each filling volume 5 mL (with both 2 and 5 mL filling volume), 10 mL and 50 mL, containing water for injection. Each analysis sequence contained the test syringes, 10 positive and 10 negative control syringes. None of the tested negative controls showed a trace of blue colour. All of the tested positive controls showed a blue colour. None of the tested syringes showed a blue colour. The validation has demonstrated the suitability of the method to detect a leakage larger or equal of 170 µm.

### Silicon

For the determination of silicon concentrations an in house developed and validated inductively coupled plasma-mass spectrometry (ICP-MS) method was used.

#### Method and material

A Thermo Scientific iCAP Qc, ICP-MS (Inductively coupled plasma-mass spectrometry) for the determination of silicon in water for injection (WFI) samples was used. Silicon has a high first ionisation potential and background polyatomic ions affect all three silicon isotopes. Silicon has three isotopes 28Si, 29Si and 30Si with respectively 92.2%, 4.68% and 3.09% relative abundances of naturally occurring isotopes and a first ionisation potential 8.2 eV. Germanium was used as internal standard and has 5 isotopes 70Ge, 72Ge, 73Ge, 74Ge and 76Ge with a first ionisation potential of 7.9 eV. The isotopes used in the method were 28Si and 73Ge with a dwell time of 0.01 s and 20 sweeps. All measurements were done in the KED Mode (Kinetic Energy Discrimination) with Helium used as collision gas to decrease the interference at mass 28Si (9N_2_^+^ and ^12^C^16^O). Data acquisition was done with Qtegra software. See online supplemental table 1 for the settings of ICP-MS method.

10.1136/ejhpharm-2020-002333.supp1 Supplementary data

ICP-MS grade 1000 ppm TraceCERT Silicon standard was obtained from Fluka analytical. Nitric acid (68%) *OPTIMA* was obtained from Fisher Chemical. ICP-MS grade 1000 ppm TraceCERT Germanium standard was obtained from Fluka analytical. Calibration and tune solutions were obtained from Thermo Scientific. Ultrapure water was produced using the Milli-Q Advantage A10 water purification system with Q-POD dispenser from Merck Millipore Corporation.

### Sample preparation

Aliquots of 2.0 mL sample were transferred to 10 mL plastic tubes and 20 µL internal standard solution (40 ppm Germanium solution) was added, vortexed and measured. We used the ICP-MS grade silicon standard and spiked in-house prepared 0.5% HNO_3_ to get a calibration curve. Five calibration standards were prepared in the range of 250 ppb to 750 ppb Pure water for injection was used as blank sample. See online supplemental figure 1 for the calibration curve.

10.1136/ejhpharm-2020-002333.supp2 Supplementary data

A factor is calculated based on the difference in molecular weight of silicon (Si3 84.26 g/mol) available in the monomer of polydimethylsiloxane (PDMS) (C_8_H_24_Si_3_O_2_ 236.53 g/mol), the used lubricant, to correlate the measured silicon concentration to the amount of released silicon oil. See online supplemental figure 2 for the molecule structure of PDMS.

10.1136/ejhpharm-2020-002333.supp3 Supplementary data

## Results

### General chemistry tests

The results of the general chemistry test for all solutions and syringes are summarised in tables 3 and 4). In general, the pH was fairly constant across all solutions tested, except for WFI and NaCl 0.9%, and was relatively unchanged (pH values did not deviate more than 0.4 units from target pH). The pH of WFI and NaCl 0.9% varied in time because no buffer capacity was present. For the lower pH ranges (pH 2–5) and higher pH ranges (pH 10–11), the pH was fairly constant during the study period (±1 pH unit). The UV data (measured between 230 nm and 360 nm) provides an indication of the level of organic extractables. For the normal pH ranges (phosphate buffer pH 5.8–8.0, NaCl 0.9%) the level of organic extractables was relatively low ≤0.03 absorbance units (Au). For the more extreme pH range (pH 2 and 11.4) the level of organic extractables was intermediate, with a maximum of 0.03 Au for phosphate buffer pH two and a maximum of 0.08 Au for phosphate buffer pH 11.4. The difference between the 5 mL syringes and the 50 mL syringes was negligible. Only the weight loss in the 5 mL syringes was slightly higher than in the 50 mL syringes, but the results were within acceptance limits.

**Table 3:** Results general chemistry tests syringe 5 mL

Solvent	Minimum- maximum level measured during 0–36 months						
	pH	Clarity	Colour	Weight loss	Transparency	Reducing substances	Absorbance (Au)
Phosphate buffer pH 2.0	2.2–2.3	Clear	<BY7	0%–0.6%	Cloudiness perceptible		<0.01–0.04
Phosphate buffer pH 5.8	5.7–5.9	Clear	<BY7	0%–0.8%	Cloudiness perceptible		<0.01–0.02
Phosphate buffer pH 8.0	7.8–7.9	Clear	<BY7	0%–0.6%	Cloudiness perceptible		<0.01–0.01
Phosphate buffer pH 11.4	11.4–11.5	Clear	<BY7	0%–0.6%	Cloudiness perceptible		0.02–0.08
NaCl 0.9%	4.0–10.0	Clear	<BY7	0%–0.7%	Cloudiness perceptible	0.1–1.2 mL	<0.01–0.02
IPA 5%	NA	Clear	<BY7	0%–0.8%	Cloudiness perceptible		<0.01–0.03

**Table 4:** Results general chemistry tests syringe 50 mL

Solvent	Minimum- maximum level measured during 0–36 months						
	pH	Clarity	Colour	Weight loss	Transparency	Reducing substances	Absorbance (Au)
Phosphate buffer pH 2.0	2.2–2.3	Clear	<BY7	0%–0.1%	Cloudiness perceptible		<0.01–0.03
Phosphate buffer pH 5.8	5.4–5.9	Clear	<BY7	0%–0.1%	Cloudiness perceptible		<0.01–0.03
Phosphate buffer pH 8.0	7.8–7.9	Clear	<BY7	0%–0.4%	Cloudiness perceptible		<0.01–0.02
Phosphate buffer pH 11.4	11.4–11.6	Clear	<BY7	0%–0.1%	Cloudiness perceptible		<0.01–0.06
NaCl 0.9%	4.7–8.9	Clear	<BY7	0%–0.1%	Cloudiness perceptible	0.2–1.3 mL	<0.01–0.02
IPA 5%	NA	Clear	<BY7	0%–0.1%	Cloudiness perceptible		0.01–0.03

### Subvisible particles

The results of the amount of subvisible particles are summarised in online supplemental tables 2 and 3. All measured numbers of particles were within the acceptance limits;≤6000 particles≥10 µm and ≤600 particles≥25 µm per syringe. The amount of particles was varied in time but no trend was visible during the extraction study or between the solvents.

10.1136/ejhpharm-2020-002333.supp4 Supplementary data

10.1136/ejhpharm-2020-002333.supp5 Supplementary data

### Sterility/closure integrity

All performed sterility tests and closure integrity tests complied with the acceptance limits.

### Silicon

**Figure 1:**
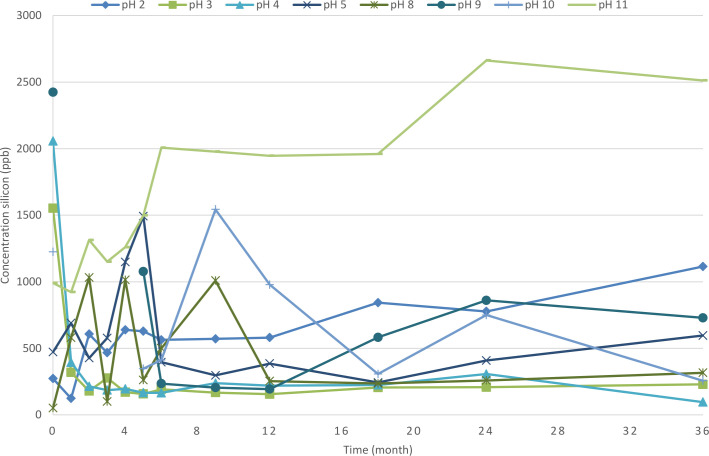
Concentration silicon (ppb) during 36 months for 5 mL syringe.

**Figure 2:**
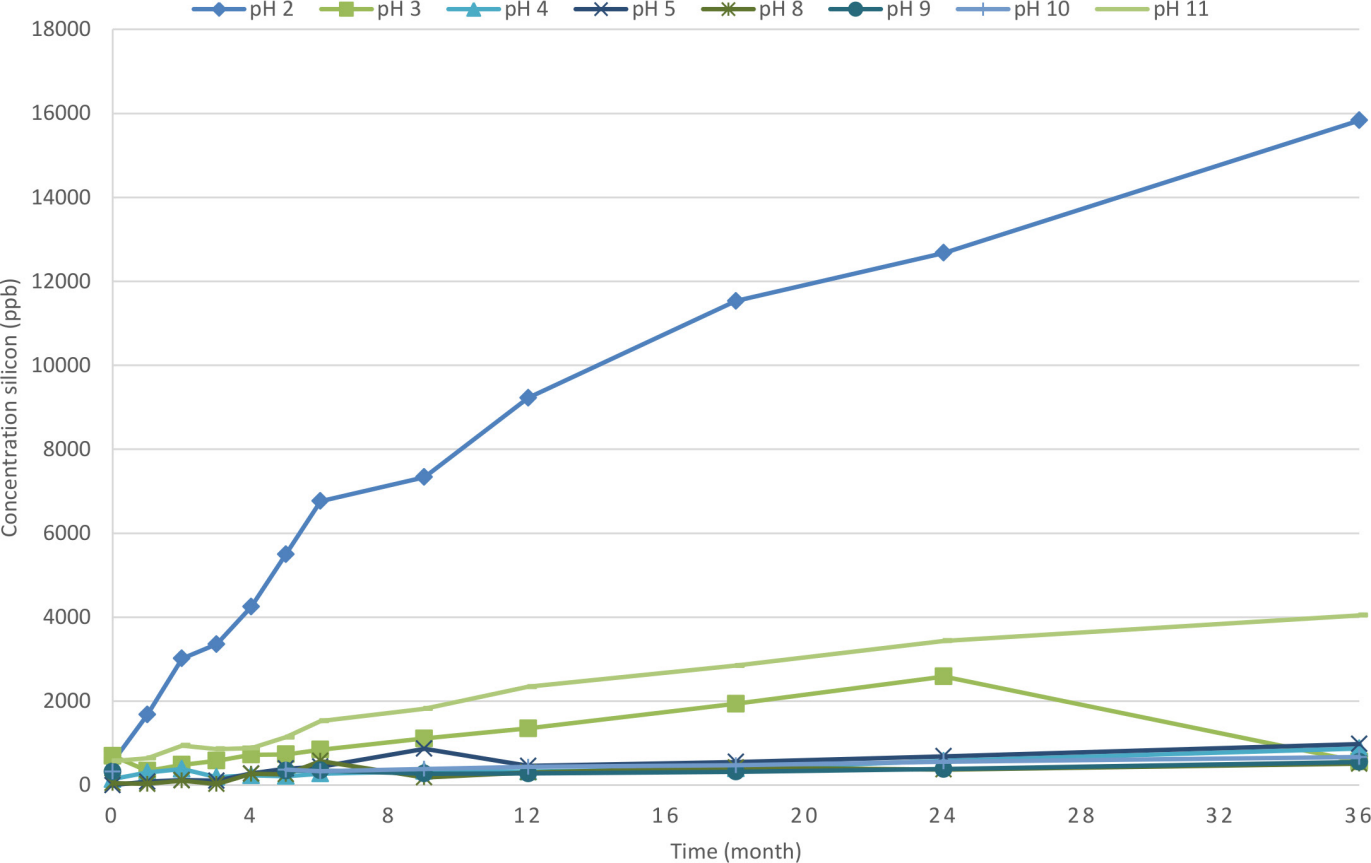
Concentration silicon (ppb) during 36 months for 50 mL syringe.

In the 5 mL and 50 mL syringes different concentrations of silicon were measured. See figures 1 and 2 for the silicon concentrations (n=2 syringes for every point in time) at each different pH-value during 36 months. The silicon concentration at t=0 was high for most solutions, after which the silicon concentration first decreased and then increased again during the further study period with the maximum value mostly after t=36 months. For the 5 mL syringe higher silicon values were found at pH 11 which increased in time up to more than 2500 ppb after 36 months. Overall higher silicon concentrations were measured for the 50 mL syringes. At the lower pH range (pH 2–3) and higher pH range (10-11) high concentration silicon were measured up to more than 4000 ppb at pH 11 after 36 months and even more than 15 000 ppb at pH 2 after 36 months.

After measuring high concentrations of silicon (>500 ppb) in the low and high pH ranged validation batches (pH 2.0 and pH 11.0) and also high concentrations in random syringes in mid-range pH batches (pH 5.0 and pH 8.0) additional experiments were conducted to investigate where the silicon originated from. The hypothesis was that the materials used in the filling process could have a negative impact on the silicon concentration. The test solution was brought into the filling assembly which consisted of a 3 L bag and silicone distribution tubing (to and from the bag). The total volume in the silicone tubing used in the filling assembly for 50 mL syringes is 201.57 mL. With no flow the silicon was extracted from the silicone tubing, causing an increase in silicon concentration in the first syringes. As soon as a constant flow is realised, the extraction of silicon from the tubing is reduced to a minimum.

To determine whether the silicon concentration in the solution is present in free silica form or as a part of silicon oil, a silicate test (Merck, 1.14792.0001) was performed. The test solution (WFI pH 2.0, 50 mL) was brought into a sulfuric acid environment in which the silicate ions could react with molybdate ions to form a yellow heteropoly acid. This is reduced to silicomolybdenum blue. The silicate concentration was measured semi-quantitatively by visual comparison of the colour of the measurement solution with the coloured fields on a colour disk. In addition to the test solution a silicon standard of approximately 5 ppm and a solution with approximately 5% of added silicon oil were tested. The 5 ppm silicon solution turned blue, where neither the diluted silicon oil nor the test solution showed any reaction, confirming the hypothesis that in the injection solutions silicon is present as an oil.

Furthermore the test solution, ultrapure water and a solution of 5% silicon oil were observed under a microscope (Zeiss AXIOSKOP, 20 x objective, 10 x ocular). The 5% silicon oil solution contained clear drops which showed different layers of depth and spheres confirming the presence of oil. Ultrapure water was clear and contained no spheres. The test solution (WFI pH 2.0, 50 mL) contained one drop that showed different layers of depth, which could be identified as oil.

The highest measured silicon value was 15 836 ppb for the 50 mL syringe with pH 2 at 36 months. This corresponds to 792 µg silicon and finally in 2.22 mg silicon oil released from a 50 mL syringe. The 50 mL syringe with pH 3, measured a maximum of 4171 ppb silicon, corresponding to 0.59 mg silicon oil released from the syringe. For the 5 mL syringe the highest concentrations silicon was 2519 ppb at pH 11. This corresponds to 0.04 mg silicon oil. In online supplemental table 4 another approach is calculated based on the maximum amount silicon which may be available in the syringe according to Ph. Eur. 3.2.8 (maximum 0.25 mg/cm2).

10.1136/ejhpharm-2020-002333.supp6 Supplementary data

## Discussion

All results from the general chemistry tests, subvisible particles, sterility and closure integrity were within the predefined acceptance limits of the qualification programme. No significant differences were observed in the absorbance, relating to possible extractables between the different solvents phosphate buffer, NaCl 0.9% and IPA 5%. Higher values for the absorbance test were found in the lower and higher pH region of the phosphate buffer solutions. This is in line with the findings of Jenke *et al* who examined the compatibility of several polymeric (including glass) and elastomeric materials with solution- based pharmaceutical products.^5^ The UV absorption profile (210–280 nm) for polypropylene was high and increased with higher pH values. The UV profile for COP and cyclic olefin copolymer (COC) were similar in the study of Jenke *et al* 5 and also to our results; higher absorption at pH 2 and pH 12 in comparison to neutral pH was found. Jenke *et al* 5 also examined the elastomers separately and found similar results to the polymeric materials; the UV absorbance of the extracts for increased at pH 12 for all elastomers. Furthermore, Jenke *et al* 5 also examined the Total Organic Carbon (TOC) levels, with low amounts of extractable TOC for glass, COP and COC. The extracted levels for COC and COP are similar and are not affected by the pH of the extracted medium. The elastomeric materials had an extracted TOC that was similar at pH 2 and neutral pH but which increased significantly at pH 12.

Glucose 5% was not included as a solvent in this study, based on our analysis of suitable products, which were mostly water soluble products with NaCl 0.9% as solvent. When glucose 5% is used as solvent, additional experiments may need to be performed.

We developed a new closure integrity test to test the syringes which included a positive control. We found that all syringes were adequately closed.

The concentration of silicon varied between the different syringes and the different solutions with highest values at pH 2 and pH 11. The silicon oil, a polydimethylsiloxane (PDMS), is applied to both stopper and barrel to achieve smooth gliding of the stopper along the barrel. PDMS is the most used lubricant for medical devices and primary packaging systems because of its stability, hydrophobicity, lubricity and low toxicity.^10^ Selected PDMS compounds have been extensively studied to assess their suitability for use in cosmetic and pharmaceutical applications. It is generally believed that data gathered from these selected polymers can be applied to all of the materials except for certain compounds of very low molecular weight. There is no indication that toxicity is related to molecular weight or viscosity of these fluids.^10^

When silicon oil migrates out of the system into the solution this may become a product quality concern because silicon oil subvisible particles may interfere with particulate matter counts and be indistinguishable from other subvisible particles.^11^ Our results showed no relation between the amount of silicon oil and the number of subvisible particles. Another issue could be that silicon oil may accelerate protein instabilities.^11^ This was not investigated in this qualification programme since we do not intend to produce protein containing injections. The last issue may be a safety aspect; when injecting, silicone-oil induced aggregates, or silicone-oil containing complexes, this may elicit immunogenic responses. In our study the highest value of silicon oil was 2.22 mg for the 50 mL syringe with pH 2 and 0.59 mg silicon oil for the 50 mL syringes with pH 3. Based on our experience in hospital pharmacy practice, products with a pH of 2 are unlikely to be produced. Products with a pH of 3 (eg, midazolam HCl) may be produced, resulting in 0.59 mg silicon. A vast amount of data is available about the toxicity of silicone oil, by different routes of administration (skin, inhalation, intraperitoneal, subcutaneous) which all show a low toxicity profile which varies from a no-observed-effect- level (NOAEL) of 50 mg/kg per day for a 28 day period, to 0.1 g/kg intravenously for 25 days without obvious ill effects in rabbits. The Dow Chemical Company has placed the intravenous LD50 for the DC 200 (350 cSt) fluid in rabbits at about 0.5 g/kg.^10 12^

Another approach is comparing our results to the maximum allowed value of 0.25 mg silicon/cm^2^ according to Ph. Eur. 3.2.8. which may be present in the syringes (see online supplemental table 4). This value is much higher than the values found in our study, implicating that more than 10 000 syringes could be administered without expecting adverse effects.

From the literature, it’s known that the rate and extent of silicone extraction in solution were highest on exposure to extreme pH solutions (pH 2 and pH 12) and are higher at higher temperatures (55°C) compared with refrigeration (5°C).^13^One of the advantage of the sterilised prefilled syringes was the fact that they can be stored at room temperature instead of in the refrigerator due to limited refrigerator capacity in the pharmacy as well as on the ward. Based on this refrigeration was not investigated in this study.

In the analysis of the selection of the available products to produce in the syringe the maximum pH was 9.3. Due to the high silicon level found in an intermediate time point of the study period (t=9 months) for pH 10 in the 5 mL syringes, maybe more experiments need to be performed to establish the suitability of the syringe for pH 10. Although this was only found in one measurement, which makes it very likely drug products with a pH up to 10 are suitable to produce in the syringe. This can be determined by stability testing of the specific drug products with pH 10.

Historically, glass and polypropylene are used as primary packaging material in hospital pharmacies in the Netherlands as in many other countries. With the advent of ready-to-administer syringes, cyclic olefin (co)polymer syringes are replacing polypropylene, because of the lower extractables and leachables and the possibility of terminal sterilisation, giving a longer shelf life to the products.

The observations in our study confirm the suitability of the COP syringe as primary packaging material. Although a full stability study is still necessary for all future products.

## Conclusions

The chosen strategy for the qualification programme provided a good overview of the possible extractables from the syringes. The cyclic olefin polymer syringes including stopper and tip cap were found to be suitable as primary packaging materials for the production of water soluble products with pH varying from 3 to 9.

**What this paper adds**

**What is already known on this subject**

- New products should undergo a validation process to show chemical and microbiological stability.
- No general monographs are available to test the compatibility of a new product in COP syringes.
- COP syringes are not used as primary packaging material in hospital pharmacy in Europe.

**What this study adds**

- A risk-based and pragmatic strategy for qualification of primary packaging material in hospital pharmacy.
- COP syringes are suitable as primary packaging material in hospital pharmacy.

## Data Availability

Data are available upon reasonable request. Data are available on request.
