# Supplementary material for: Science- and risk-based strategy to qualify prefillable autoclavable syringes as primary packaging material
Source: Eur J Hosp Pharm. 2021 Jan 27;29(5):248–54. doi: 10.1136/ejhpharm-2020-002333 (PMC9660587; doi:10.1136/ejhpharm-2020-002333)
Supplement: Supplementary data [file ejhpharm-2020-002333supp001.pdf]

Supplemental table 1: Settings IPC-MS silicon method.

Parameter	Value
Pump tubing	PVC peristaltic pump tubing, id 0.51 mm, Orange/Yellow
Peristaltic pump speed	40 rpm
Nebulizer	PFA-ST
Interface cones	Nickel
RF Power	1550 W
Cool gas flow	14 L/min
Auxiliary gas flow	0.8 L/min
Nebulizer gas flow	1.19 L/min
Collision gas flow	4.7 L/min
Collision gas	Helium
Injector type	Quartz
Injector ID	2.5 mm
Measurement mode	KED (Kinetic Energy Discrimination)
KED barrier voltage	3 Volt
Autosampler	SC 2 DX Elemental Scientific Inc.
