# Supplementary figures and images for: Science- and risk-based strategy to qualify prefillable autoclavable syringes as primary packaging material
Source: Eur J Hosp Pharm. 2021 Jan 27;29(5):248–54. doi: 10.1136/ejhpharm-2020-002333 (PMC9660587; doi:10.1136/ejhpharm-2020-002333)

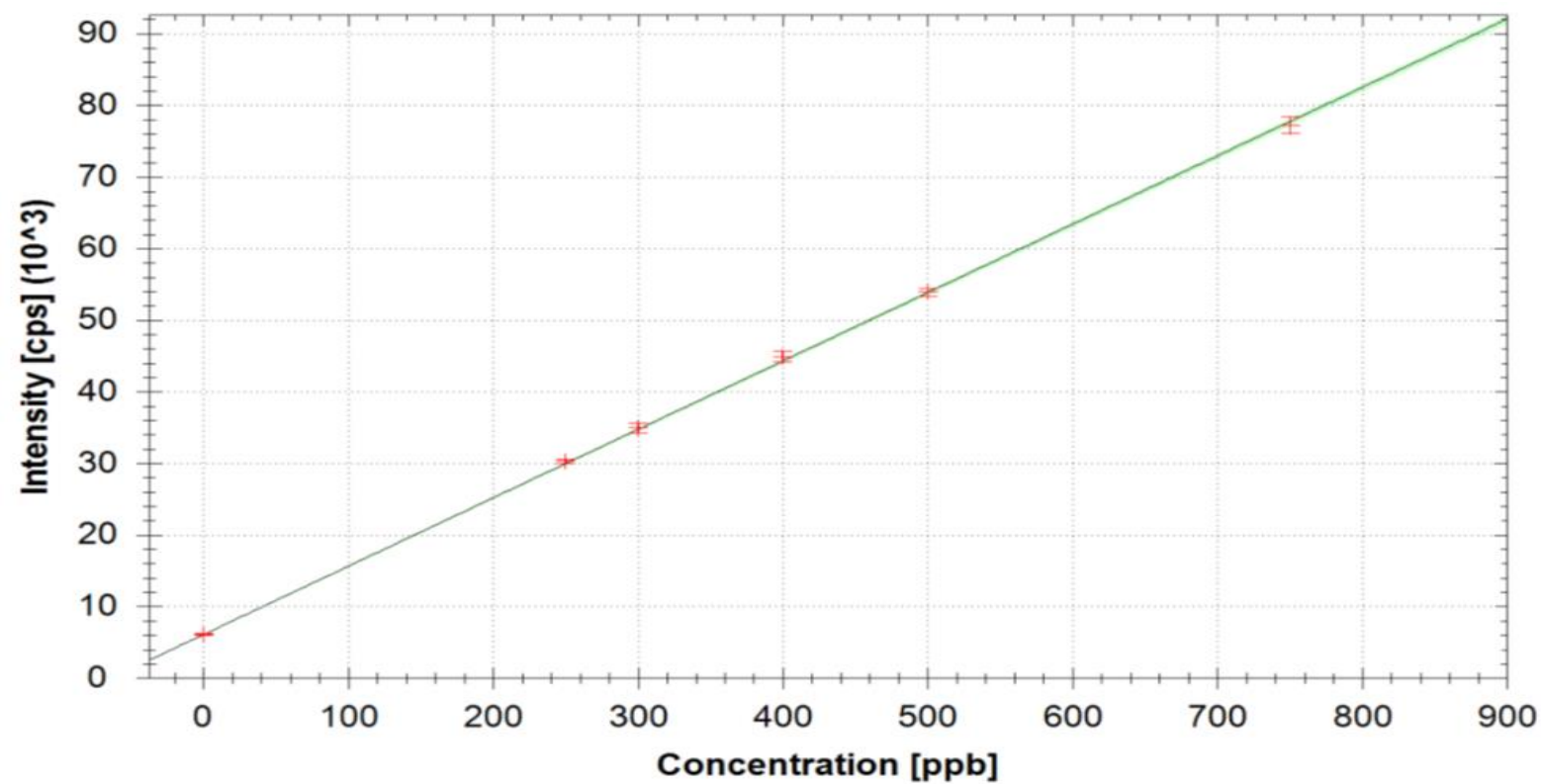

Supplemental Figure 1: calibration curve silicon method.  $f(x) = 95.5357 \cdot x + 6064.5831$ ,  $R^2 = 0.9997$ , BEC = 63.480 ppb, LoD = 2.9079 ppb.

Supplement: Supplementary data [file ejhpharm-2020-002333supp002.pdf]
