# Supplementary material for: Science- and risk-based strategy to qualify prefillable autoclavable syringes as primary packaging material
Source: Eur J Hosp Pharm. 2021 Jan 27;29(5):248–54. doi: 10.1136/ejhpharm-2020-002333 (PMC9660587; doi:10.1136/ejhpharm-2020-002333)
Supplement: Supplementary data [file ejhpharm-2020-002333supp003.pdf]

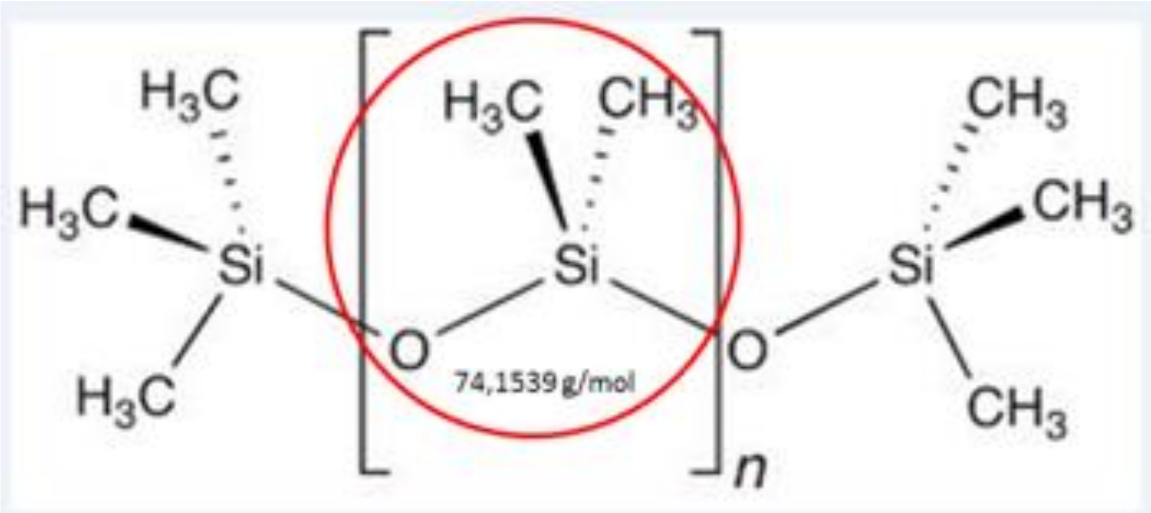

Supplemental Figure 2: Molecule structure PDMS, n represents the relative number of repeating siloxane units in the polymer chain.
