# Supplementary material for: Science- and risk-based strategy to qualify prefillable autoclavable syringes as primary packaging material
Source: Eur J Hosp Pharm. 2021 Jan 27;29(5):248–54. doi: 10.1136/ejhpharm-2020-002333 (PMC9660587; doi:10.1136/ejhpharm-2020-002333)
Supplement: Supplementary data [file ejhpharm-2020-002333supp004.pdf]

Supplemental table 2: Results subvisible particles (bracketing scheme). Number of particles presented in 5mL syringe.

	T= 0		T= 3		T= 6		T= 9		T= 12		T=18		T=24		T=36	
	≥ 10 µm	≥ 25 µm	≥ 10 µm	≥ 25 µm	≥ 10 µm	≥ 25 µm	≥ 10 µm	≥ 25 µm	≥ 10 µm	≥ 25 µm	≥ 10 µm	≥ 25 µm	≥ 10 µm	≥ 25 µm	≥ 10 µm	≥ 25 µm
Phosphate buffer pH 2.0	631	16	135	10							315	**10**			287	7
Phosphate buffer pH 5.8	46	16							230	6					307	4
Phosphate buffer pH 8.0	338	9			78	2							571	20	162	4
Phosphate buffer pH 11.4	329	3					64	1							402	5
NaCl 0.9%	88	2	89	4			66	3					155	2	242	8
IPA 5%	43	1					179	6							133	2
WFI pH 2.0	221	2	162	3	273	8	145	1	616	13					403	11
WFI pH 3.0	231	3	293	2	1649	24									229	3
WFI pH 4.0	262	2	357	9	911	4									96	0
WFI pH 5.0	300	11	164	8	257	2	697	17	269	7			658	18	166	2
WFI pH 8.0	157	3	215	4	232	5	198	2	341	5			293	14	311	6
WFI pH 9.0					106	1	130	2	134	0			430	4	124	3
WFI pH 10.0					80	0	280	7	143	1			339	4	153	4
WFI pH 11.0	481	10	430	4	479	7	242	2	670	16			1287	12	693	6
