# Supplementary material for: Science- and risk-based strategy to qualify prefillable autoclavable syringes as primary packaging material
Source: Eur J Hosp Pharm. 2021 Jan 27;29(5):248–54. doi: 10.1136/ejhpharm-2020-002333 (PMC9660587; doi:10.1136/ejhpharm-2020-002333)
Supplement: Supplementary data [file ejhpharm-2020-002333supp005.pdf]

BMJ Publishing Group Limited (BMJ) disclaims all liability and responsibility arising from any reliance placed on this supplemental material which has been supplied by the author(s)

Supplemental table 3: Results subvisible particles (bracketing scheme). Number of particles presented in 50mL syringe.

	**T= 0**		**T= 3**		**T= 6**		**T= 9**		**T= 12**		**T=18**		**T=24**		**T=36**	
	**≥ 10 µm**	**≥ 25 µm**	**≥ 10 µm**	**≥ 25 µm**	**≥ 10 µm**	**≥ 25 µm**	**≥ 10 µm**	**≥ 25 µm**	**≥ 10 µm**	**≥ 25 µm**	**≥ 10 µm**	**≥ 25 µm**	**≥ 10 µm**	**≥ 25 µm**	**≥ 10 µm**	**≥ 25 µm**
Phosphate buffer pH 2.0	103	10	57	0							57	**9**			146	1
Phosphate buffer pH 5.8	557	13							97	8					242	3
Phosphate buffer pH 8.0	207	0			533	13							183	0	629	7
Phosphate buffer pH 11.4	257	20					1140	33							1409	30
NaCl 0.9%	560	107	127	20			260	10					500	0	1045	7
IPA 5%	532	20					713	50							584	4
WFI pH 2.0	37	3	247	10	173	0	553	7	120	0					83	10
WFI pH 3.0	80	3	88	3	127	0									558	2
WFI pH 4.0	139	6	63	4	205	1									874	5
WFI pH 5.0	1287	10	1467	3	297	10	150	10	1193	3			567	3	1950	30
WFI pH 8.0	200	0	667	27	133	7	483	10	267	0			1020	23	280	13
WFI pH 9.0					3827	17	3719	25	2886	12			4547	35	3396	31
WFI pH 10.0					3429	4	2764	10	2592	13			3694	34	2053	30
WFI pH 11.0	857	7	187	7	1107	17	2347	63	913	3			1963	13	3390	37
