# Supplementary material for: Science- and risk-based strategy to qualify prefillable autoclavable syringes as primary packaging material
Source: Eur J Hosp Pharm. 2021 Jan 27;29(5):248–54. doi: 10.1136/ejhpharm-2020-002333 (PMC9660587; doi:10.1136/ejhpharm-2020-002333)
Supplement: Supplementary data [file ejhpharm-2020-002333supp006.pdf]

Supplemental table 4: Calculation silicon oil based on 0.25mg/cm^2 according to Ph. Eur. 3.2.8.

Volume (mL)	Height of the graduation (cm)	Intern surface (cm²)	Upper limit of allowed silicon oil (mg/ syringe)	Max amount of Silicon per syringe (mg) at the worst case* measured	Max amount of Silicon per syringe (µg/L) at the worst case* measured
5	4.284	16.41	4.10	1.46	292212
50	9.031	75.33	18.83	6.71	134166

* Based on:

Si_3	84.26 g/mol
$C_8H_{24}Si_3O_2$ (PDMS)	236.53 g/mol
Factor	2.8073
